# The genome sequence of the Lunar Marbled Brown, *Drymonia ruficornis *(Hufnagel, 1766)

**DOI:** 10.12688/wellcomeopenres.21225.1

**Published:** 2024-04-17

**Authors:** William B. V. Langdon, Lucy M. Morley, University of Oxford and Wytham Woods Genome Acquisition Lab, Darwin Tree of Life Barcoding collective, Wellcome Sanger Institute Tree of Life Management, Samples and Laboratory team, Wellcome Sanger Institute Scientific Operations: Sequencing Operations, Wellcome Sanger Institute Tree of Life Core Informatics team, Tree of Life Core Informatics collective, Darwin Tree of Life Consortium

**Affiliations:** 1 Department of Biologu, University of Oxford, Oxford, England, UK

**Keywords:** Drymonia ruficornis, Lunar Marbled Brown moth, genome sequence, chromosomal, Lepidoptera

## Abstract

We present a genome assembly from an individual male *Drymonia ruficornis* (the Lunar Marbled Brown; Arthropoda; Insecta; Lepidoptera; Notodontidae). The genome sequence is 369.6 megabases in span. Most of the assembly is scaffolded into 29 chromosomal pseudomolecules, including the Z sex chromosome. The mitochondrial genome has also been assembled and is 17.11 kilobases in length.

## Species taxonomy

Eukaryota; Opisthokonta; Metazoa; Eumetazoa; Bilateria; Protostomia; Ecdysozoa; Panarthropoda; Arthropoda; Mandibulata; Pancrustacea; Hexapoda; Insecta; Dicondylia; Pterygota; Neoptera; Endopterygota; Amphiesmenoptera; Lepidoptera; Glossata; Neolepidoptera; Heteroneura; Ditrysia; Obtectomera; Noctuoidea; Notodontidae; Notodontinae; *Drymonia*; *Drymonia ruficornis* (Hufnagel, 1766) (NCBI:txid988178).

## Background

The Lunar Marbled Brown ( *Drymonia ruficornis*) is a moth of the Notodontidae family ( Waring *et al.*, 2017). It has a wingspan of 35–40mm, and the forewings are shades of grey with a whitish middle section edged in black wavy lines, although there is a rare darker form ( Waring *et al.*, 2017). It appears similar to the marbled brown ( *D. dodonea*), however *D. ruficornis* has a conspicuous black crescent in the white area which is characteristic ( Skinner & Wilson, 2009; Waring *et al.*, 2017). Its hindwings are an unmarked smoky grey. Adults are on the wing in April and May, slightly earlier than *D. dodonea* ( Waring *et al*., 2017). Both sexes come to light and the species has one generation per year ( Skinner & Wilson, 2009). Larvae of the lunar marbled brown are bluish green with yellow subdorsal and spiracular stripes. They feed on oaks ( *Quercus* spp.) between late May to late July then overwinter as a pupa just below the ground’s surface ( Henwood *et al.*, 2020). The species inhabits deciduous woodland, but may sometimes be found in hedgerows, gardens, and parks ( Waring *et al*., 2017).

The Lunar Marbled Brown is resident in Britain and has increased in abundance in recent decades ( Conrad *et al.*, 2006). It is relatively common in southern England, Wales and Ireland, but more localised in northern England and Scotland ( Waring *et al*., 2017). Globally, it is distributed across Europe up to southern Scandinavia and Asia Minor ( GBIF Secretariat, 2024). *D. ruficornis* may be split into subspecies, with *D. ruficornis ruficornis* in Europe, the darker ssp. *concolor* in Turkey, Lebanon and Israel, and ssp. *vivida* in southern Spain and north Africa ( Schintlmeister, 2008). *D. ruficornis* was present in a high proportion (93.8%) of bat faecal samples from Czech forests, possibly consumed at both adult and caterpillar stages ( Blažek *et al.*, 2021).

## Genome sequence report

**Figure 1.**
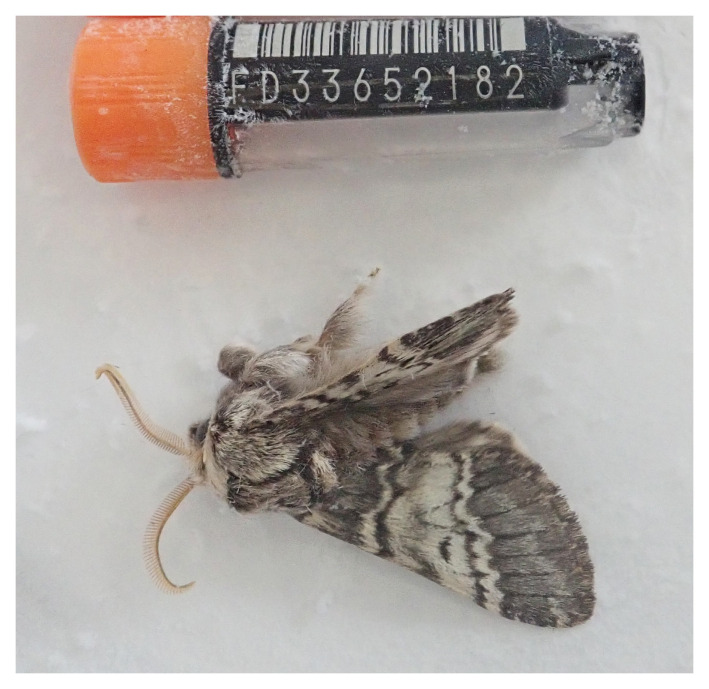
Photograph of the *Drymonia ruficornis* (ilDryRufi1) specimen used for genome sequencing.

The genome was sequenced from one male *Drymonia ruficornis* ( Figure 1) collected from Wytham Woods, Oxfordshire, UK (51.78, –1.32). A total of 50-fold coverage in Pacific Biosciences single-molecule HiFi long reads was generated. Primary assembly contigs were scaffolded with chromosome conformation Hi-C data. Manual assembly curation corrected 11 missing joins or mis-joins and removed two haplotypic duplications, reducing the scaffold number by 6.06%.

**Figure 2.**
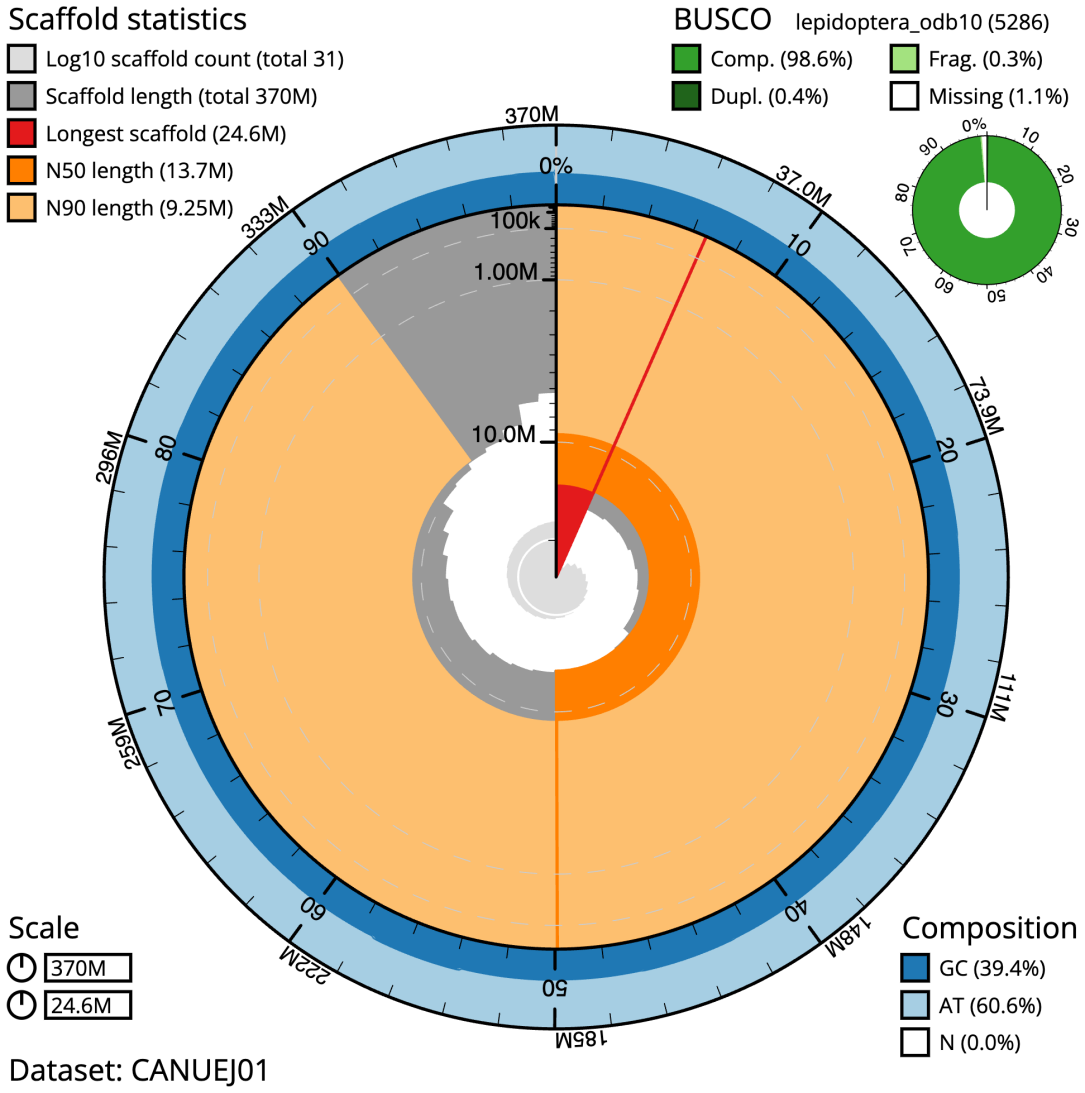
Genome assembly of *Drymonia ruficornis*, ilDryRufi1.1: metrics. The BlobToolKit snail plot shows N50 metrics and BUSCO gene completeness. The main plot is divided into 1,000 size-ordered bins around the circumference with each bin representing 0.1% of the 369,611,541 bp assembly. The distribution of scaffold lengths is shown in dark grey with the plot radius scaled to the longest scaffold present in the assembly (24,596,455 bp, shown in red). Orange and pale-orange arcs show the N50 and N90 scaffold lengths (13,723,929 and 9,248,621 bp), respectively. The pale grey spiral shows the cumulative scaffold count on a log scale with white scale lines showing successive orders of magnitude. The blue and pale-blue area around the outside of the plot shows the distribution of GC, AT and N percentages in the same bins as the inner plot. A summary of complete, fragmented, duplicated and missing BUSCO genes in the lepidoptera_odb10 set is shown in the top right. An interactive version of this figure is available at https://blobtoolkit.genomehubs.org/view/CANUEJ01/dataset/CANUEJ01/snail.

**Figure 3.**
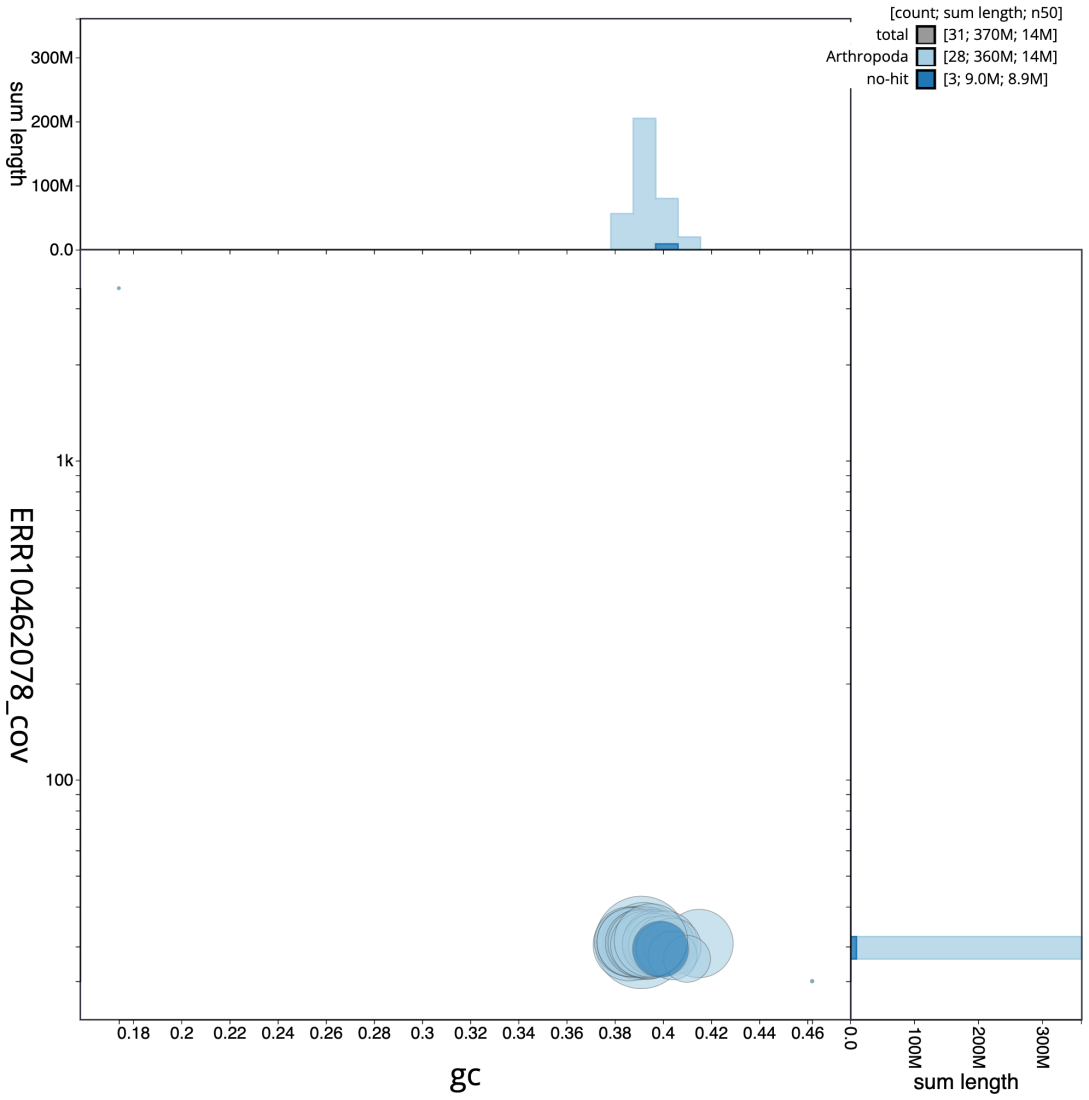
Genome assembly of *Drymonia ruficornis*, ilDryRufi1.1: BlobToolKit GC-coverage plot. Sequences are coloured by phylum. Circles are sized in proportion to sequence length. Histograms show the distribution of sequence length sum along each axis. An interactive version of this figure is available at https://blobtoolkit.genomehubs.org/view/CANUEJ01/dataset/CANUEJ01/blob.

**Figure 4.**
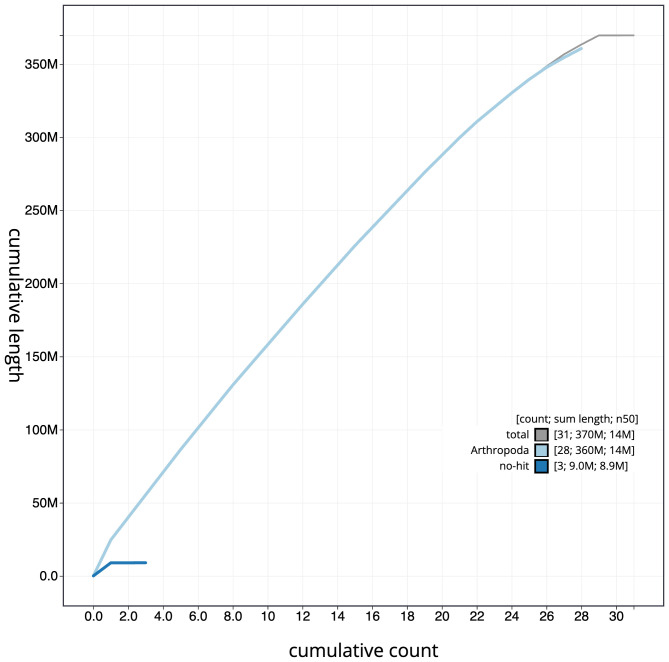
Genome assembly of *Drymonia ruficornis*, ilDryRufi1.1: BlobToolKit cumulative sequence plot. The grey line shows cumulative length for all sequences. Coloured lines show cumulative lengths of sequences assigned to each phylum using the buscogenes taxrule. An interactive version of this figure is available at https://blobtoolkit.genomehubs.org/view/CANUEJ01/dataset/CANUEJ01/cumulative.

**Figure 5.**
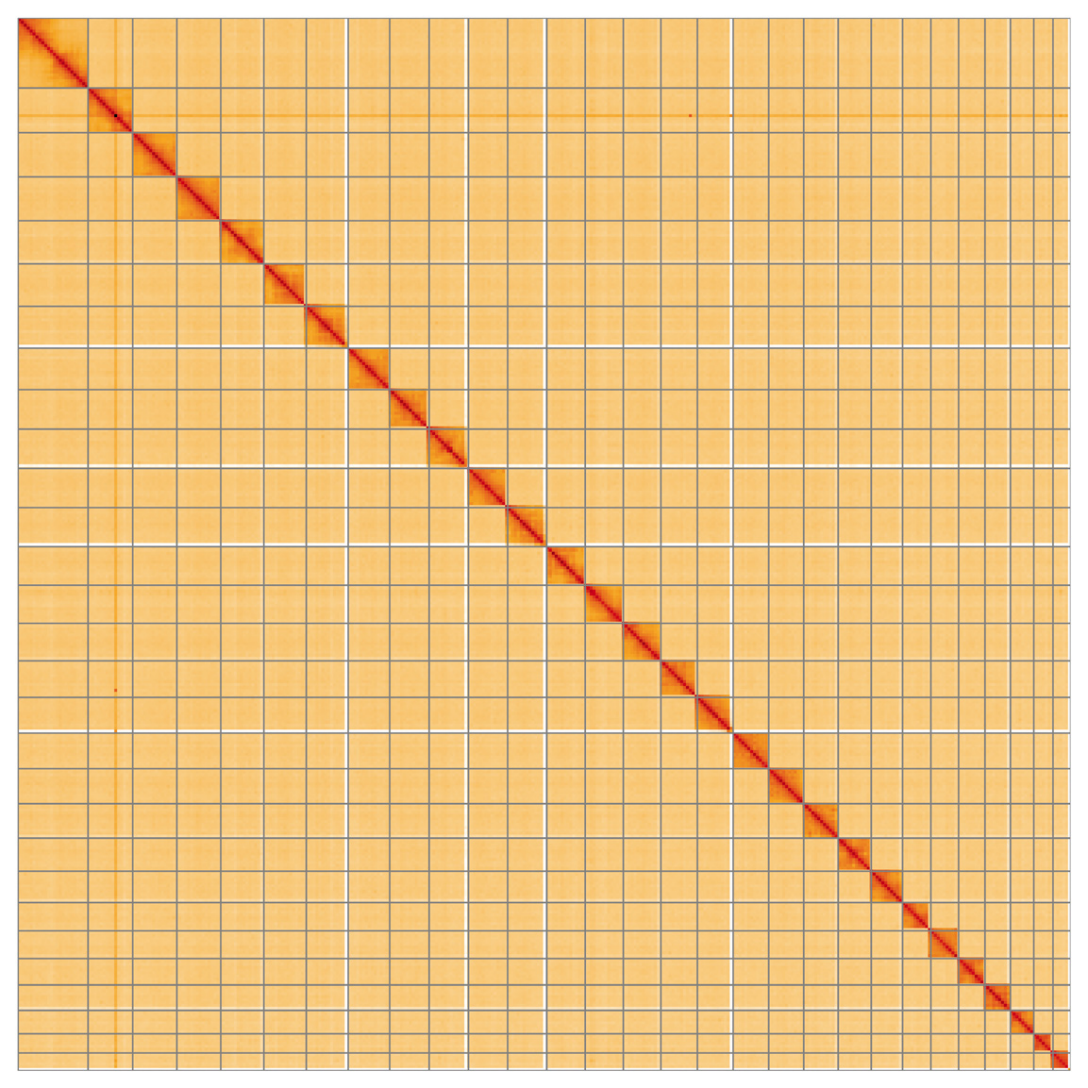
Genome assembly of *Drymonia ruficornis*, ilDryRufi1.1: Hi-C contact map of the ilDryRufi1.1 assembly, visualised using HiGlass. Chromosomes are shown in order of size from left to right and top to bottom. An interactive version of this figure may be viewed at https://genome-note-higlass.tol.sanger.ac.uk/l/?d=T-KnTvFzSIe0DmVNr7UmSw.

The final assembly has a total length of 369.6 Mb in 30 sequence scaffolds with a scaffold N50 of 13.7 Mb ( Table 1). The snail plot in Figure 2 provides a summary of the assembly statistics, while the distribution of assembly scaffolds on GC proportion and coverage is shown in Figure 3. The cumulative assembly plot in Figure 4 shows curves for subsets of scaffolds assigned to different phyla. Most (99.98%) of the assembly sequence was assigned to 29 chromosomal-level scaffolds, representing 28 autosomes and the Z sex chromosome. Chromosome-scale scaffolds confirmed by the Hi-C data are named in order of size ( Figure 5; Table 2). While not fully phased, the assembly deposited is of one haplotype. Contigs corresponding to the second haplotype have also been deposited. The mitochondrial genome was also assembled and can be found as a contig within the multifasta file of the genome submission.

**Table 1.** Genome data for *Drymonia ruficornis*, ilDryRufi1.1.

Project accession data		
Assembly identifier	ilDryRufi1.1	
Species	*Drymonia ruficornis*	
Specimen	ilDryRufi1	
NCBI taxonomy ID	988178	
BioProject	PRJEB57276	
BioSample ID	SAMEA110451594	
Isolate information	ilDryRufi1: thorax (DNA sequencing), head (Hi-C sequencing), abdomen (RNA sequencing)	
Assembly metrics *		*Benchmark*
Consensus quality (QV)	64.4	*≥ 50*
*k*-mer completeness	100.0%	*≥ 95%*
BUSCO **	C:98.6%[S:98.2%,D:0.4%], F:0.3%,M:1.1%,n:5,286	*C ≥ 95%*
Percentage of assembly mapped to chromosomes	99.98%	*≥ 95%*
Sex chromosomes	Z	*localised * *homologous pairs*
Organelles	Mitochondrial genome: 17.11 kb	*complete single * *alleles*
Raw data accessions		
PacificBiosciences SEQUEL II	ERR10462078	
Hi-C Illumina	ERR10466812	
PolyA RNA-Seq Illumina	ERR11606291	
Genome assembly		
Assembly accession	GCA_947859195.1	
*Accession of alternate * *haplotype*	GCA_947859005.1	
Span (Mb)	369.6	
Number of contigs	96	
Contig N50 length (Mb)	6.5	
Number of scaffolds	30	
Scaffold N50 length (Mb)	13.7	
Longest scaffold (Mb)	24.6	

*Assembly metric benchmarks are adapted from column VGP-2020 of “Table 1: Proposed standards and metrics for defining genome assembly quality” from Rhie *et al.* (2021). **BUSCO scores based on the lepidoptera_odb10 BUSCO set using version 5.3.2. C = complete [S = single copy, D = duplicated], F = fragmented, M = missing, n = number of orthologues in comparison. A full set of BUSCO scores is available at https://blobtoolkit.genomehubs.org/view/CANUEJ01/dataset/CANUEJ01/busco.

**Table 2.** Chromosomal pseudomolecules in the genome assembly of *Drymonia ruficornis*, ilDryRufi1.

INSDC accession	Chromosome	Length (Mb)	GC%
OX401904.1	1	24.6	39.0
OX401905.1	2	15.69	39.0
OX401906.1	3	15.5	39.0
OX401908.1	4	15.13	39.5
OX401909.1	5	14.9	38.5
OX401910.1	6	14.7	39.0
OX401911.1	7	14.57	39.5
OX401912.1	8	13.86	38.5
OX401913.1	9	13.78	38.5
OX401914.1	10	13.77	39.5
OX401915.1	11	13.72	38.5
OX401916.1	12	13.52	39.0
OX401917.1	13	13.36	41.5
OX401918.1	14	13.18	39.5
OX401919.1	15	12.82	39.0
OX401920.1	16	12.55	39.0
OX401921.1	17	12.5	39.0
OX401922.1	18	12.29	39.5
OX401923.1	19	12.09	40.0
OX401924.1	20	11.6	40.0
OX401925.1	21	11.02	39.5
OX401926.1	22	9.93	40.0
OX401927.1	23	9.74	40.0
OX401928.1	24	9.25	40.5
OX401929.1	25	8.94	40.0
OX401930.1	26	8.2	40.0
OX401931.1	27	6.74	40.5
OX401932.1	28	6.22	41.0
OX401907.1	Z	15.41	39.5
OX401933.1	MT	0.02	18.0

The estimated Quality Value (QV) of the final assembly is 64.4 with *k*-mer completeness of 100.0%, and the assembly has a BUSCO v5.3.2 completeness of 98.6% (single = 98.2%, duplicated = 0.4%), using the lepidoptera_odb10 reference set ( *n* = 5,286).

Metadata for specimens, barcode results, spectra estimates, sequencing runs, contaminants and pre-curation assembly statistics are given at https://links.tol.sanger.ac.uk/species/988178.

## Methods

### Sample acquisition and nucleic acid extraction

A *Drymonia ruficornis* (specimen ID Ox002148, ToLID ilDryRufi1) was collected from Wytham Woods, Oxfordshire (biological vice-county Berkshire), UK (latitude 51.78, longitude –1.32) on 2022-04-25 by potting. The specimen was collected and identified by William Langdon (University of Oxford) and preserved on dry ice.

The workflow for high molecular weight (HMW) DNA extraction at the Wellcome Sanger Institute (WSI) includes a sequence of core procedures: sample preparation; sample homogenisation, DNA extraction, fragmentation, and clean-up. In sample preparation, the ilDryRufi1 sample was weighed and dissected on dry ice ( Jay *et al.*, 2023). Tissue from the thorax was homogenised using a PowerMasher II tissue disruptor ( Denton *et al.*, 2023a).

HMW DNA was extracted in the WSI Scientific Operations core using the Automated MagAttract v2 protocol ( Oatley *et al.*, 2023). The DNA was sheared into an average fragment size of 12–20 kb in a Megaruptor 3 system with speed setting 31 ( Bates *et al.*, 2023). Sheared DNA was purified by solid-phase reversible immobilisation ( Strickland *et al.*, 2023): in brief, the method employs a 1.8X ratio of AMPure PB beads to sample to eliminate shorter fragments and concentrate the DNA. The concentration of the sheared and purified DNA was assessed using a Nanodrop spectrophotometer and Qubit Fluorometer and Qubit dsDNA High Sensitivity Assay kit. Fragment size distribution was evaluated by running the sample on the FemtoPulse system.

RNA was extracted from abdomen tissue of ilDryRufi1 in the Tree of Life Laboratory at the WSI using the RNA Extraction: Automated MagMax™ *mir*Vana protocol ( do Amaral *et al.*, 2023). The RNA concentration was assessed using a Nanodrop spectrophotometer and a Qubit Fluorometer using the Qubit RNA Broad-Range Assay kit. Analysis of the integrity of the RNA was done using the Agilent RNA 6000 Pico Kit and Eukaryotic Total RNA assay.

Protocols developed by the WSI Tree of Life laboratory are publicly available on protocols.io ( Denton *et al.*, 2023b).

### Sequencing

Pacific Biosciences HiFi circular consensus DNA sequencing libraries were constructed according to the manufacturers’ instructions. Poly(A) RNA-Seq libraries were constructed using the NEB Ultra II RNA Library Prep kit. DNA and RNA sequencing was performed by the Scientific Operations core at the WSI on Pacific Biosciences SEQUEL II (HiFi) and Illumina NovaSeq 6000 (RNA-Seq) instruments. Hi-C data were also generated from head tissue of ilDryRufi1 using the Arima2 kit and sequenced on the Illumina NovaSeq 6000 instrument.

### Genome assembly, curation and evaluation

Assembly was carried out with Hifiasm ( Cheng *et al.*, 2021) and haplotypic duplication was identified and removed with purge_dups ( Guan *et al.*, 2020). The assembly was then scaffolded with Hi-C data ( Rao *et al.*, 2014) using YaHS ( Zhou *et al.*, 2023). The assembly was checked for contamination and corrected as described previously ( Howe *et al.*, 2021). Manual curation was performed using HiGlass ( Kerpedjiev *et al.*, 2018) and Pretext ( Harry, 2022). The mitochondrial genome was assembled using MitoHiFi ( Uliano-Silva *et al.*, 2023), which runs MitoFinder ( Allio *et al.*, 2020) or MITOS ( Bernt *et al.*, 2013) and uses these annotations to select the final mitochondrial contig and to ensure the general quality of the sequence.

A Hi-C map for the final assembly was produced using bwa-mem2 ( Vasimuddin *et al.*, 2019) in the Cooler file format ( Abdennur & Mirny, 2020). To assess the assembly metrics, the *k*-mer completeness and QV consensus quality values were calculated in Merqury ( Rhie *et al.*, 2020). This work was done using Nextflow ( Di Tommaso *et al.*, 2017) DSL2 pipelines “sanger-tol/readmapping” ( Surana *et al.*, 2023a) and “sanger-tol/genomenote” ( Surana *et al.*, 2023b). The genome was analysed within the BlobToolKit environment ( Challis *et al.*, 2020) and BUSCO scores ( Manni *et al.*, 2021; Simão *et al.*, 2015) were calculated.

Table 3 contains a list of relevant software tool versions and sources.

**Table 3.** Software tools: versions and sources.

Software tool	Version	Source
BlobToolKit	4.2.1	https://github.com/blobtoolkit/blobtoolkit
BUSCO	5.3.2	https://gitlab.com/ezlab/busco
Hifiasm	0.16.1-r375	https://github.com/chhylp123/hifiasm
HiGlass	1.11.6	https://github.com/higlass/higlass
Merqury	MerquryFK	https://github.com/thegenemyers/MERQURY.FK
MitoHiFi	2	https://github.com/marcelauliano/MitoHiFi
PretextView	0.2	https://github.com/wtsi-hpag/PretextView
purge_dups	1.2.3	https://github.com/dfguan/purge_dups
sanger-tol/genomenote	v1.0	https://github.com/sanger-tol/genomenote
sanger-tol/readmapping	1.1.0	https://github.com/sanger-tol/readmapping/tree/1.1.0
YaHS	1.1a.2	https://github.com/c-zhou/yahs

### Wellcome Sanger Institute – Legal and Governance

The materials that have contributed to this genome note have been supplied by a Darwin Tree of Life Partner. The submission of materials by a Darwin Tree of Life Partner is subject to the **‘Darwin Tree of Life Project Sampling Code of Practice’**, which can be found in full on the Darwin Tree of Life website here. By agreeing with and signing up to the Sampling Code of Practice, the Darwin Tree of Life Partner agrees they will meet the legal and ethical requirements and standards set out within this document in respect of all samples acquired for, and supplied to, the Darwin Tree of Life Project. 

Further, the Wellcome Sanger Institute employs a process whereby due diligence is carried out proportionate to the nature of the materials themselves, and the circumstances under which they have been/are to be collected and provided for use. The purpose of this is to address and mitigate any potential legal and/or ethical implications of receipt and use of the materials as part of the research project, and to ensure that in doing so we align with best practice wherever possible. The overarching areas of consideration are:

•     Ethical review of provenance and sourcing of the material

•     Legality of collection, transfer and use (national and international) 

Each transfer of samples is further undertaken according to a Research Collaboration Agreement or Material Transfer Agreement entered into by the Darwin Tree of Life Partner, Genome Research Limited (operating as the Wellcome Sanger Institute), and in some circumstances other Darwin Tree of Life collaborators.

## Data Availability

European Nucleotide Archive: *Drymonia ruficornis* (lunar marbled brown). Accession number PRJEB57276; https://identifiers.org/ena.embl/PRJEB57276 ( Wellcome Sanger Institute, 2023). The genome sequence is released openly for reuse. The *Drymonia ruficornis* genome sequencing initiative is part of the Darwin Tree of Life (DToL) project. All raw sequence data and the assembly have been deposited in INSDC databases. The genome will be annotated using available RNA-Seq data and presented through the Ensembl pipeline at the European Bioinformatics Institute. Raw data and assembly accession identifiers are reported in Table 1.

## Author information

Members of the University of Oxford and Wytham Woods Genome Acquisition Lab are listed here: https://doi.org/10.5281/zenodo.7125292.

Members of the Darwin Tree of Life Barcoding collective are listed here: https://doi.org/10.5281/zenodo.4893703.

Members of the Wellcome Sanger Institute Tree of Life Management, Samples and Laboratory team are listed here: https://doi.org/10.5281/zenodo.10066175.

Members of Wellcome Sanger Institute Scientific Operations: Sequencing Operations are listed here: https://doi.org/10.5281/zenodo.10043364.

Members of the Wellcome Sanger Institute Tree of Life Core Informatics team are listed here: https://doi.org/10.5281/zenodo.10066637.

Members of the Tree of Life Core Informatics collective are listed here: https://doi.org/10.5281/zenodo.5013541.

Members of the Darwin Tree of Life Consortium are listed here: https://doi.org/10.5281/zenodo.4783558.
