# Supplementary material for: The genome sequence of the Lunar Marbled Brown, Drymonia ruficornis (Hufnagel, 1766)
Source: Wellcome Open Res. 2024 Apr 17;9:201. [Version 1] doi: 10.12688/wellcomeopenres.21225.1 (PMC11474161; doi:10.12688/wellcomeopenres.21225.1)
Supplement: Peer review history

# Reviewer report: Reviewer response for version 1

**DOI:** 10.21956/wellcomeopenres.23472.r101868

**Published:** 2024-10-14

**Authors:** Ariel D Chipman

**Affiliations:** 1 Ecology, Evolution & Behavior, Hebrew University of Jerusalem, Jerusalem, Jerusalem District, Israel

This is a fairly standard report on the sequencing of a new insect genome. All the necessary information for evaluating the quality of the genome is included within the article. Methods are clearly explained and are what is to be expected off this type of report. Basic information about the biology off the species is given.

Are sufficient details of methods and materials provided to allow replication by others?

Yes

Is the rationale for creating the dataset(s) clearly described?

Yes

Are the datasets clearly presented in a useable and accessible format?

Yes

Are the protocols appropriate and is the work technically sound?

Yes

Reviewer Expertise:

Arthropod evolution. Comparative genomics.

I confirm that I have read this submission and believe that I have an appropriate level of expertise to confirm that it is of an acceptable scientific standard.

# Reviewer report: Reviewer response for version 1

**DOI:** 10.21956/wellcomeopenres.23472.r90373

**Published:** 2024-08-12

**Authors:** Hermes E. Escalona

**Affiliations:** 1 Australian National Insect Collection, CSIRO, Canberra, Australia

The genome of the Lunar Marbled Brown ( *Drymonia ruficornis*):  the results  of this work are clear and well explained. In addition, the introduction provided comparative information on the species that can be valuable in population, taxonomy and similar studies. The results are accessible publicly and the methodology is well described.

Are sufficient details of methods and materials provided to allow replication by others?

Yes

Is the rationale for creating the dataset(s) clearly described?

Yes

Are the datasets clearly presented in a useable and accessible format?

Yes

Are the protocols appropriate and is the work technically sound?

Yes

Reviewer Expertise:

Entomology

I confirm that I have read this submission and believe that I have an appropriate level of expertise to confirm that it is of an acceptable scientific standard.
